# Structural Basis for the Regulation of PPARγ Activity by Imatinib

**DOI:** 10.3390/molecules24193562

**Published:** 2019-10-01

**Authors:** Jun Young Jang, Hyun-Jung Kim, Byung Woo Han

**Affiliations:** 1 Research Institute of Pharmaceutical Sciences, College of Pharmacy, Seoul National University, Seoul 08826, Korea; nosvc4@snu.ac.kr; 2 College of Pharmacy, Chung-Ang University, Seoul 06974, Korea; hyunjungkim@cau.ac.kr

**Keywords:** imatinib, PPARγ, antidiabetic drug, type 2 diabetes, crystal structure

## Abstract

Imatinib is an effective anticancer drug for the treatment of leukemia. Interestingly, when an FDA-approved drug library was tested for agents that block peroxisome proliferator-activated receptor γ (PPARγ) phosphorylation at Ser245 to evaluate possibilities of antidiabetic drug repositioning, imatinib was determined as a PPARγ antagonist ligand. However, it is not well understood how imatinib binds to PPARγ or would improve insulin sensitivity without classical agonism. Here, we report the crystal structure of the PPARγ R288A mutant in complex with imatinib. Imatinib bound to Arm2 and Arm3 regions in the ligand-binding domain (LBD) of PPARγ, of which the Arm3 region is closely related to the inhibition of PPARγ phosphorylation at Ser245. The binding of imatinib in LBD induced a stable conformation of helix H2′ and the Ω loop compared with the ligand-free state. In contrast, imatinib does not interact with Tyr473 on PPARγ helix H12, which is important for the classical agonism associated with side effects. Our study provides new structural insights into the PPARγ regulation by imatinib and may contribute to the development of new antidiabetic drugs targeting PPARγ while minimizing known side effects.

## 1. Introduction

Peroxisome proliferator-activated receptor γ (PPARγ) belongs to the thyroid hormone receptor-like nuclear receptor subfamily 1, which is one of the ligand-activated transcription factors [1]. PPARγ forms a heterodimer with retinoid X receptors (RXRs), recruits coactivators, and then binds to the cognate peroxisome proliferative response elements on target genes [2]. Through this process, PPARγ regulates the transcription of target genes, which plays an important role in adipocyte differentiation, lipid metabolism, glucose homeostasis, insulin sensitization, and inflammation [3,4]. Thus, PPARγ is a good therapeutic target for type 2 diabetes mellitus, as well as other metabolic diseases including obesity and atherosclerosis [5].

Compared with other nuclear receptors, PPARγ contains a considerably larger Y-shaped ligand-binding pocket (LBP) with a volume of 1300–1440 Å^3^ and is known to be activated by numerous endogenous and synthetic ligands [6,7,8]. Types of synthetic PPARγ ligands are typically represented as full agonists, partial agonists, and antagonists. PPARγ full agonists induce a conformational change of helix H12 via hydrogen bond networks with Tyr473 of helix H12, His323 of helix H5, and His449 of helix H10 to recruit coactivators, thereby resulting in strong adipogenesis-related transcriptional agonism [6,9]. As PPARγ full agonists, thiazolidinediones (TZDs), such as rosiglitazone and pioglitazone, have been widely used in treating type 2 diabetes mellitus due to their potent insulin-sensitizing effects [8]. However, TZDs have been prescribed with caution due to their known side effects, including weight gain, fluid retention, increased adipogenesis, and bone loss [9,10]. Compared with PPARγ full agonist, PPARγ partial agonists bind differently to the ligand-binding domain (LBD) of PPARγ, which preferentially stabilize the four-stranded β-sheet and helix H3, but leave helix H12 in a very dynamic state [11,12]. PPARγ partial agonists have diminished agonist efficacy compared with full agonists, but may retain excellent insulin-sensitizing effects without causing side effects as much as full agonists [11,12]. In addition, PPARγ partial agonists may induce a differential recruitment of beneficial coactivators associated with regulating glucose metabolism, energy expenditure, and adipocyte differentiation [13]. Thus, PPARγ partial agonists are another class of compounds that activate PPARγ with sufficient insulin-sensitizing efficacy, accompanied by few side effects.

A major challenge in developing safer antidiabetic compounds targeting PPARγ is to maintain the advantageous insulin-sensitizing effects of PPARγ ligands while minimizing these undesirable side effects. In 2010, the phosphorylation of PPARγ at Ser245 (in PPARγ1; Ser273 in PPARγ2) by cyclin-dependent kinase 5 (Cdk5) was shown to be linked to insulin resistance [14]. Cdk5-mediated phosphorylation of PPARγ does not affect its classical transcriptional activity; however, it can lead to impairment in the regulation of genes involved in insulin sensitivity such as adiponectin and adipsin [14]. Various synthetic PPARγ ligands such as full agonists, partial agonists, and antagonists have been shown to induce graded PPARγ transcriptional agonism, yet they effectively block the phosphorylation of PPARγ at Ser245 to similar degrees [11,14]. Recently, structural studies revealed that the alternate binding site and the hydrophobic region between helix H3 and the four-stranded β-sheet of PPARγ could affect PPARγ phosphorylation at Ser245 [15,16]. Thus, other ligands selectively targeting these regions would be potential candidates as a potent antidiabetic drug with reduced side effects.

**Figure 1:**
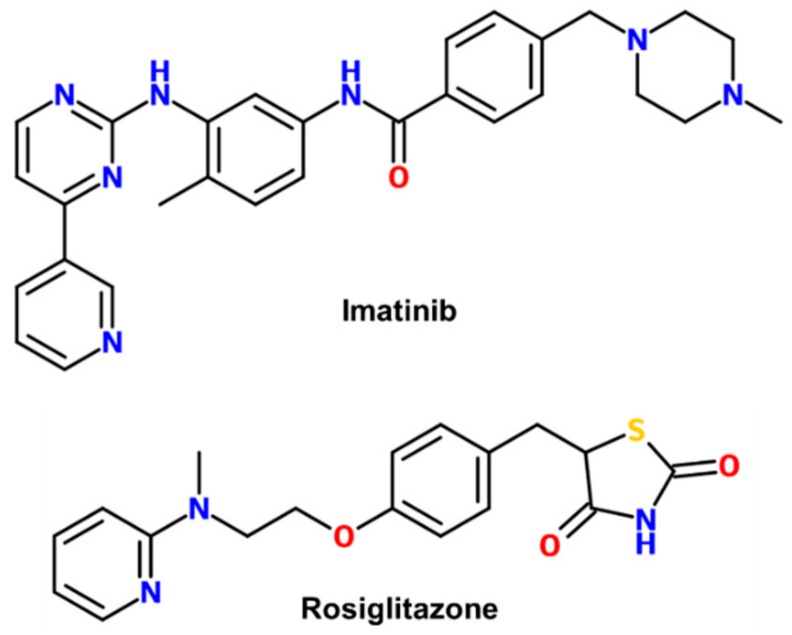
Chemical structures of imatinib and rosiglitazone.

Imatinib is a well-known anticancer drug to treat chronic myeloid leukemia (CML) and acute lymphoblastic leukemia (ALL) that are Philadelphia chromosome-positive, some gastrointestinal stromal tumors, and other cancer-related syndromes [17,18,19]. Imatinib is the first-generation drug of a BCR-ABL tyrosine kinase inhibitor (Figure 1) [20]. Interestingly, imatinib was recently identified as a PPARγ antagonist ligand that improves insulin sensitivity without classical agonism from a drug repositioning screening of an FDA-approved drug library [21]. Imatinib directly bound to PPARγ and inhibited the Cdk5-mediated phosphorylation of PPARγ in a dose-dependent manner [21]. However, its exact binding mode as a PPARγ phosphorylation site inhibitor has not been revealed. Here, we report the crystal structure of PPARγ R288A mutant in complex with imatinib.

## 2. Results

### 2.1. Overall Structure of Imatinib-Bound PPARγ R288A Mutant LBD

**Figure 2:**
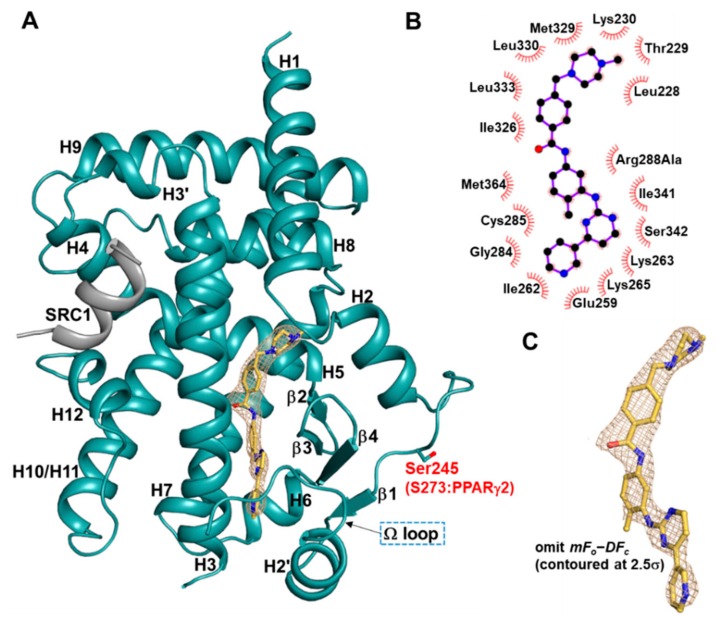
Overall structure of imatinib-bound PPARγ R288A mutant LBD. (**A**) Ribbon diagram of imatinib-bound PPARγ R288A mutant LBD (light teal) with the helical LxxLL motif-containing peptide (grey). Imatinib molecule and the Cdk5-mediated phosphorylation site Ser245 are shown in yellow orange and light teal stick models, respectively. The electron density for imatinib in the *mFo–DFc* omit map is displayed as a wheat teal-colored mesh (contoured at 2.5σ). (**B**) Schematic diagram of the interactions between PPARγ R288A mutant LBD and imatinib, as calculated using LigPlot+ [23]. Hydrophobic effects are indicated by atoms with spokes and spoked arcs. Carbon, nitrogen, and oxygen atoms are colored in black, blue, and red, respectively. (**C**) A magnified image of bound imatinib in a stick model with the *mFo–DFc* omit electron density map (contoured at 2.5σ).

Imatinib is a 2-phenylaminopyrimidine derivative that is chemically distinct from the thiazolidinedione (TZD) class of PPARγ full agonists (Figure 1). To gain insights into the binding mode of PPARγ phosphorylation site inhibitor imatinib, we determined the crystal structure of the PPARγ R288A mutant LBD in complex with imatinib in the presence of a peptide derived from human steroid receptor coactivator-1 (SRC-1) at 2.75 Å resolution. We were unable to resolve the crystal structure of imatinib-bound PPARγ wild-type (WT) LBD. However, from previously reported PPARγ LBD structures, we found that Arg288 was very flexible depending on ligands. More interestingly, PPARγ R288H mutation was observed in colon cancer patients and inhibited the binding of endogenous ligands [22]. To reduce the effect of Arg288 on ligand binding, we generated the PPARγ R288A mutant LBD construct and could resolve the imatinib-bound PPARγ R288A mutant LBD structure. Crystals of imatinib-bound PPARγ R288A mutant LBD belonged to the *orthorhombic* space group *P*2_1_2_1_2 and contained one monomer in an asymmetric unit. The overall structure resembled LBDs of the other nuclear receptors with 13 α-helices and a mixed four-stranded β-sheet (Figure 2A). The Cdk5-mediated phosphorylation site Ser245 of PPARγ LBD was located on the loop between helix H2 and strand β1. The C-terminal helix H12 was folded over the LBP in an active conformation. Helices H3, H4, and H12 surrounded a shallow hydrophobic groove, which was the binding site for the helical LxxLL motif of the SRC-1 coactivator.

### 2.2. Imatinib Occupies Arm2 and Arm3 Regions in PPARγ R288A Mutant LBD

**Figure 3:**
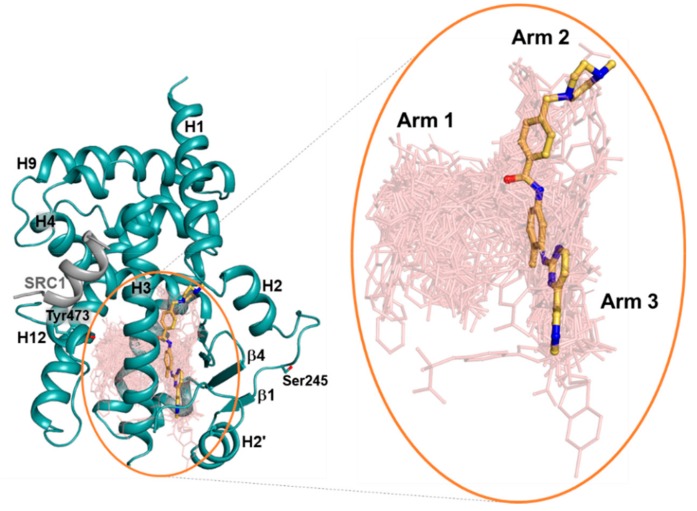
Superposition of imatinib and other reported ligands from known PPARγ complex structures. In total, 144 ligand-bound PPARγ LBD structures in the protein data bank (PDB) were superimposed onto the imatinib-bound PPARγ R288A mutant LBD structure (ribbon in light teal). Yellow orange stick model indicates imatinib and salmon lines represent the other ligands. Tyr473 of helix H12 and the Cdk5-mediated phosphorylation site Ser245 are shown in light teal stick models. Right panel shows a close-up view of PPARγ LBP, with the Arm1, Arm2, and Arm3 regions of PPARγ LBP labeled.

In the structure of imatinib-bound PPARγ R288A mutant LBD, we observed a clear extra electron density calculated from the final refined model. The omit map could be modeled explicitly with imatinib (Figure 2C). To compare the binding mode of imatinib with other PPARγ ligands, we superimposed 144 structures of PPARγ complexed with many different ligands onto the imatinib-bound PPARγ R288A mutant LBD. Superposition of many other ligands indicated that Arm1, Arm2, and Arm3 regions were formed in the PPARγ LBP, of which imatinib occupied the Arm2 and Arm3 regions (Figure 3). The primary interaction between imatinib and PPARγ R288A mutant LBD was hydrophobic effects (Figure 2B). The benzamide ring and N-methylpiperazine ring of imatinib were in contact with a pocket in the Arm2 region, consisting of residues from the H1–H2 loop (Leu228, Thr229, and Lys230) and helix H5 (Ile326, Met329, Leu330, and Leu333). The methylbenzene ring and pyridylpyrimidine moiety of imatinib were surrounded by residues from helix H2′ (Glu259), Ω loop (Ile262, Lys263, and Lys265), helix H3 (Gly284, Cys285, and Arg288Ala), strand β3 (Ile341 and Ser342), and helix H7 (Met364) in the Arm3 region. Imatinib did not occupy the Arm1 region and had no interaction with the residue Tyr473 of helix H12, which is commonly presented in the TZD-bound PPARγ (Figure 3). In general, the binding mode of the imatinib molecule in complex with kinase proteins adopted two conformations. First, imatinib bound to the ABL kinase (Protein Data Bank (PDB) ID: 1IEP), adopting a trans-conformation with the methylbenzene and benzamide rings trans to the pyridylpyrimidine moiety (Figure S1) [24]. In the structure of the imatinib-bound Syk kinase (PDB ID: 1XBB), imatinib bound in a cis conformation, with the methylbenzene and benzamide rings cis to the pyridylpyrimidine moiety (Figure S1) [25]. The binding mode of the imatinib molecule in complex with the PPARγ R288A mutant LBD resembled the trans conformation in the imatinib-ABL complex structure (Figure 2).

### 2.3. Imatinib Binding Induced Conformational Change of H9-H10 Loop

**Figure 4:**
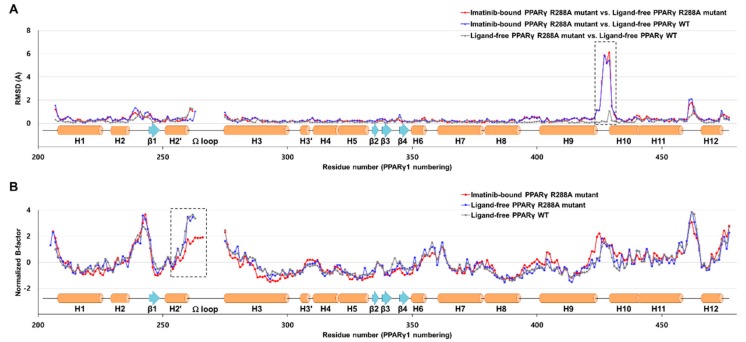
Comparison of imatinib-bound PPARγ R288A mutant LBD, ligand-free PPARγ R288A mutant LBD, and ligand-free PPARγ wild-type (WT) LBD structures with respect to Cα RMSD and normalized B-factors. (**A**) Comparison of the Cα root-mean-square deviation (RMSD) values for the imatinib-bound PPARγ R288A mutant LBD structure against the ligand-free PPARγ R288A mutant LBD and ligand-free PPARγ WT LBD (PDB ID: 6JQ7) structures. Red and blue lines represent the RMSD values for imatinib-bound PPARγ R288A mutant LBD vs. ligand-free PPARγ R288A mutant LBD and imatinib-bound PPARγ R288A mutant LBD vs. ligand-free PPARγ WT LBD structures, respectively. Grey lines represent the Cα RMSD values for ligand-free PPARγ R288A mutant LBD vs. ligand-free PPARγ WT LBD structures. Secondary structural elements are represented along the residue numbers. The H9–H10 loop region between residues Asn424 and Leu431 exhibiting a large conformational change is marked by a black-dashed box. (**B**) Comparison of the normalized B-factors for imatinib-bound PPARγ R288A mutant LBD, ligand-free PPARγ R288A mutant LBD, and ligand-free PPARγ WT LBD structures. The normalized B-factors for imatinib-bound PPARγ R288A mutant LBD, ligand-free PPARγ R288A mutant LBD, and ligand-free PPARγ WT LBD structures are represented in red, blue, and grey lines, respectively. Helix H2′ and the Ω loop, which exhibited enhanced thermal stabilities in the imatinib-bound PPARγ R288A mutant LBD structure, is marked by a black-dashed box.

**Figure 5:**
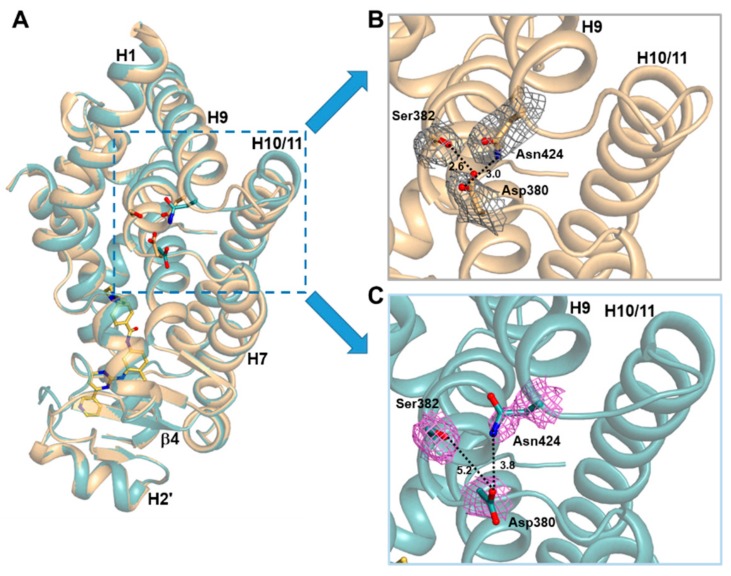
Side views of imatinib-bound and ligand-free PPARγ R288A mutant LBD structures. (**A**) Superposition of ligand-free (light orange) and imatinib-bound (light teal) PPARγ R288A mutant LBD structures. The region with a large conformational change is represented by a cyan-dashed box. (**B**) A magnified ribbon diagram of the H9–H10 loop region in the ligand-free PPARγ R288A mutant LBD structure. The residues Asp380, Ser382, and Asn424 are shown in stick models with *2mFo–DFc* electron density maps (in grey; contoured at 1.0σ). Dashed lines represent the distance between residues, and the corresponding distances (Å) are labeled. (**C**) A magnified ribbon diagram of the H9–H10 loop region in the imatinib-bound PPARγ R288A mutant LBD structure. The residues are represented in the same way as the ligand-free structure (A) with the violet-colored electron density maps.

To further gain structural insight into imatinib, we also determined the crystal structure of the ligand-free PPARγ R288A mutant LBD in the presence of the SRC-1 peptide. The overall structures of ligand-free PPARγ WT LBD (PDB ID: 6JQ7) and ligand-free PPARγ R288A mutant LBD were not significantly different, with root-mean-square deviation (RMSD) of 0.27 Å for 259 Cα atoms. When the Cα RMSD values were compared for ligand-free PPARγ WT LBD vs. imatinib-bound PPARγ R288A mutant LBD and ligand-free PPARγ R288A mutant LBD vs. imatinib-bound PPARγ R288A mutant LBD, we observed a large conformational change between residues Asn424 and Leu431 located in the H9–H10 loop (Figure 4A and Figure 5A). In the ligand-free states of PPARγ WT LBD and PPARγ R288A mutant LBD, the Oδ atoms of Asp380 formed a hydrogen bonding network with the Oγ atom of Ser382 and the Nδ atom of Asn424 with a distance of 2.6 Å and 3.0 Å, respectively (Figure 5B and Figure S2). However, when imatinib bound to PPARγ R288A mutant LBD, the electron densities of residues Asp380 and Asn424 were weakened, and the hydrogen bonding network disappeared (Figure 5C). Thus, the binding of imatinib to PPARγ LBD increased the side chain flexibility of Asp380, resulting in a conformational change of this region.

### 2.4. Imatinib Binding in PPARγ LBD Stabilizes Helix H2′ and Ω Loop

**Figure 6:**
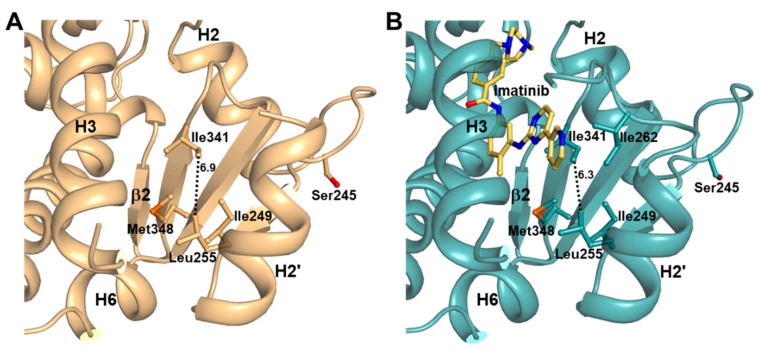
The hydrophobic interaction network in Arm3 region of PPARγ LBD. (**A**) The ligand-free PPARγ R288A mutant LBD structure is shown in a ribbon diagram (light orange), and the residues forming the hydrophobic interaction network are represented by stick models. Dashed line represents the distance between Leu255 and Ile341, and the corresponding distances (Å) are labeled. (**B**) The imatinib-bound PPARγ R288A mutant LBD structure is shown in a ribbon diagram (light teal). Imatinib is shown in a yellow orange stick model. All other marks are shown similarly to (A).

To investigate changes in protein dynamics of PPARγ LBD caused by imatinib binding, we compared normalized B-factors of the structures (Figure 4B). The B-factors of the structures were normalized to have a zero mean distribution and unit variance based on the mean value and standard deviation of the distribution of observed B-factors, as previously reported [26]. We observed that PPARγ LBD was flexible in the H2–β1 loop, the region around the Ω loop, and the H11–H12 loop due to the inherent characteristics of PPARγ LBD. The most distinctive structural difference in imatinib binding, as determined by B-factor comparative analysis, was the stability of the helix H2′ and the Ω loop regions relative to the ligand-free state. The four-stranded β-sheet region was also slightly stabilized by imatinib binding. As shown in Figure 6, we found that the distance between the Cδ atom of Ile341 and the Cδ atom of Leu255 changed from 6.9 Å in the ligand-free states to 6.3 Å in the imatinib-bound structure (Figure S3). These results showed that imatinib binding enhanced the hydrophobic interaction network of Ile249, Leu255, Ile341, and Met348 in the Arm3 region. Moreover, Ile262 of the Ω loop was also stabilized by imatinib binding and was newly involved in this hydrophobic interaction network (Figure 6B). The Ω loop of ligand-free PPARγ WT LBD and R288A mutant LBD structures could not be modeled due to missing electron density (Figure 4B and Figure 6A). However, the Ω loop, up to residue His266, could be observed in the imatinib-bound structure (Figure 4B and Figure 6B).

### 2.5. Imatinib has Higher Binding Affinity for PPARγ R288A Mutant LBD than PPARγ WT LBD

**Figure 7:**
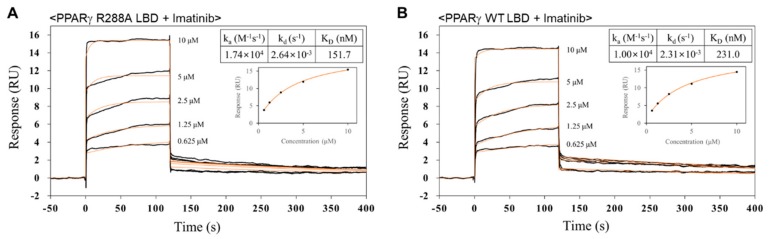
Surface plasmon resonance (SPR) analyses of the binding affinities for imatinib of PPARγ WT LBD and PPARγ R288A mutant LBD. SPR sensorgrams for imatinib binding of PPARγ R288A mutant LBD (**A**) and PPARγ WT LBD (**B**) are shown. Sensorgrams show binding at increasing concentrations (0.625, 1.25, 2.50, 5.00, and 10.0 µM) of imatinib to immobilized PPARγ R288A mutant LBD and PPARγ WT LBD. The calculated *Kd* values are also shown.

To confirm whether imatinib can interact with PPARγ WT LBD and PPARγ R288A mutant LBD, the binding affinities of imatinib for PPARγ WT LBD and PPARγ R288A mutant LBD were measured by surface plasmon resonance (SPR) analysis. SPR analysis was performed using a Biacore T200 apparatus to measure real-time interactions between imatinib molecules and PPARγ LBD proteins coupled to sensor chips in constant flow. PPARγ WT LBD and PPARγ R288A mutant LBD were immobilized on the CM5 sensor chip in an immobilization level of approximately 7000 response units (RU). For controls, reference cells were deactivated with ethanolamine (no immobilized PPARγ LBD). Imatinib was injected over the chips at concentrations ranging from 0.625 to 10 µM. The sensorgram for the interaction of imatinib with PPARγ R288A mutant LBD is shown in Figure 7A. The SPR response data were then fit to the simple bimolecular 1:1 Langmuir isotherm binding model using Biacore T200 evaluation software 3.0 (GE Healthcare). According to these data, PPARγ R288A mutant LBD showed rapid association and dissociation rates with imatinib molecules and an equilibrium binding constant (*K_d_*) of 152 nM (Figure 7A). We also detected the binding response of PPARγ WT LBD for imatinib with an equilibrium binding constant of 231 nM using the same experimental approach (Figure 7B). SPR analysis showed that PPARγ R288A mutant LBD had a stronger binding affinity for imatinib than PPARγ WT LBD. In a comparative experiment, the equilibrium binding constant of PPARγ WT LBD for rosiglitazone was 32 nM, similar to the previously reported binding constant (Figure S4) [27].

## 3. Discussion

TZDs, such as rosiglitazone and pioglitazone, are potent insulin sensitizers for the treatment of type 2 diabetes mellitus [8]. However, TZD treatment has been shown to exhibit severe side effects, including weight gain, fluid retention, and bone fracture [9,10]. While there have been numerous trials to produce a novel therapeutic agent targeting PPARγ that maintains its insulin-sensitizing effects without side effects, recent studies have shown the molecular mechanism underlying the antidiabetic effects of PPARγ agonists [14,15,16,28,29]. PPARγ phosphorylation at Ser245 by Cdk5 does not change classical PPARγ transcriptional agonism; conversely, it dysregulates the expression of insulin-sensitizing adipokines such as adipsin and adiponectin [14]. Thus, discovering compounds that block PPARγ phosphorylation at Ser245 and lack classical agonism with high binding affinity to PPARγ could be a very good strategy for the development of antidiabetic agents. Recently, Choi et al. identified that imatinib, a well-known anticancer drug, selectively blocked CDK5-mediated PPARγ phosphorylation via a drug-repositioning screening [21]. Drug repositioning is an attractive approach for the application of existing drugs to new therapeutic opportunities that can minimize the costs and risks of new drug development due to the ready availability of safety, pharmacological activity, and toxicity profiles [30]. Although the antidiabetic effect of imatinib with PPARγ has been identified, its structural basis remains unknown. Thus, we determined the structure of imatinib-bound PPARγ to more fully understand the interaction of imatinib with PPARγ and confirm the exact binding mode of imatinib in PPARγ.

In the imatinib-bound structure, the methylbenzene ring and pyridylpyrimidine moiety of imatinib occupy the Arm3 region in PPARγ LBD, which is surrounded by helix H2′, the Ω loop, and the four-stranded β-sheet (Figure 3). Recently, Filho et al. suggested an indirect mechanism of conformational change of the Ile341 side chain by ligand binding to form a hydrophobic interaction network and stabilize the helix H2′, blocking the interaction between Cdk5 and PPARγ and causing the impairment of Cdk5-mediated phosphorylation [31]. Additionally, we found that imatinib binding strengthened the hydrophobic interaction network comprised of Ile249, Leu255, Ile262, Ile341, and Met348 in the Arm3 region and stabilized the surrounding helix H2′, the Ω loop, and the four-stranded β-sheet (Figure 4B and Figure 6). In our previous study, we also reported that the *p*-methoxyphenol group of lobeglitazone stabilized the hydrophobic pocket near the alternate binding site, which is formed by Ile249, Leu255, Ile281, Ile341, and Met348 [8]. Moreover, other studies have shown that selective PPARγ modulators such as 2-BABAs and amorfrutins, which have an antidiabetic effect, can bind to this hydrophobic pocket [32,33]. Based on these results, it would appear that enhancement of the hydrophobic interaction network in the hydrophobic pocket of the Arm3 region, which consists of Ile249, Leu255, Ile262, Ile341, and Met348, by ligand binding affects inhibition of Cdk5-mediated phosphorylation of PPARγ. Therefore, it is believed that imatinib binding to PPARγ has an antidiabetic effect through this mechanism.

Interestingly, imatinib blocked the Cdk5-mediated phosphorylation of PPARγ but did not induce transcriptional activity of PPARγ, which is related to the side effects of PPARγ agonists [21]. Further, imatinib binding did not alter the conformational dynamics of helix H12 in hydrogen/deuterium exchange mass spectrometry (HDX-MS) experiments, suggesting that imatinib has no interaction with helix H12, which is associated with classical agonism [21]. Consistent with HDX-MS data, our structural analysis showed that imatinib did not occupy the Arm1 region and had no interaction with the residue Tyr473 of helix H12, which is important for transcriptional activity of PPARγ (Figure 3). In addition, imatinib deeply bound to the Arm2 region of PPARγ, causing a large structural change between residues Asn424 and Gln430 in the H9–H10 loop, which was probably first discovered through our structural data (Figure 5). This structural change of PPARγ is thought to affect binding with the heterodimer partner RXRα and reduce transcriptional activity of PPARγ. Indeed, the crystal structure of PPARγ–RXRα DNA complex showed that Ser429 and Gln430 in the H9–H10 loop of PPARγ were involved in the interaction between PPARγ LBD and RXRα LBD [34]. Taken together, these two reasons may be the cause of the lack of classical transcriptional activity of PPARγ by imatinib action.

Nevertheless, our imatinib binding mode has a limitation because we were only able to obtain the crystal structure using PPARγ R288A mutant LBD, not WT LBD. The overall structures of WT LBD (PDB ID: 6JQ7) and R288A mutant LBD in the ligand-free state were almost identical with the Cα RMSD of 0.27 Å (Figure 4A) [35]. In this respect, it is predicted that the binding mode of imatinib in PPARγ WT LBD may not show much difference from the imatinib binding mode in PPARγ R288A mutant LBD of this study. Moreover, we confirmed that imatinib had a similar level of strong binding affinity for PPARγ WT LBD (*K_d_* = 231 nM) and PPARγ R288A mutant LBD (*K_d_* = 153 nM) through the SPR binding assays (Figure 7). Interestingly, imatinib exhibited slightly better binding affinity in PPARγ R288A mutant LBD than in WT LBD, which seemed to weaken imatinib binding due to the repulsive force resulting from the close proximity between the imatinib molecule and the side chain of Arg288 (Figure S5). These additional experiments may suggest that our imatinib binding mode is nearly identical to that of PPARγ WT LBD.

In conclusion, we have shown that imatinib binding strengthens the hydrophobic interaction network and stabilizes the surrounding helix H2′, the Ω loop, and the four-stranded β-sheet. This confirms the mechanism of ligand binding in the hydrophobic pocket of the Arm3 region affects the inhibition of Cdk5-mediated phosphorylation of PPARγ. In addition, our study provides a structural explanation as to why imatinib binding does not induce classical transcriptional activity of PPARγ. We expect our structural study to shed light on the development of a new generation of PPARγ ligands as antidiabetic drugs.

## 4. Materials and Methods

### 4.1. Cloning, Expression, and Mutagenesis

The cloning, expression, purification, and crystallization of human PPARγ LBD were mainly performed as previously reported [29]. In brief, the construct of human PPARγ WT LBD (residues 195–477 in PPARγ1 numbering) was amplified from a human cDNA clone encoding PPARγ (clone ID: hMU000317) by PCR, which was provided from the Korea Human Gene Bank, Medical Genomics Research Center, KRIBB and cloned into the expression vector pET-28b(+) (Novagen, Darmstadt, Germany) with a 21-residue N-terminal fusion tag (MGSSHHHHHH SSGLVPRGSHM). The resulting recombinant PPARγ WT LBD protein contained a thrombin cleavage site in front of the starting residue Ala195, which was overexpressed in *Escherichia coli* Rosetta 2(DE3) strain. A mutation of human PPARγ WT LBD at Arg288 (R288A) was generated using the QuikChange Site-Directed Mutagenesis Kit (Stratagene, Santa Clara, CA, USA) and the mutation was confirmed by DNA sequencing.

### 4.2. Purification

The recombinant PPARγ WT LBD cells were cultured in the Luria–Bertani medium containing 30 μg/mL kanamycin at 37 °C until the mid-log phase. After the addition of 0.5 mM isopropyl β-d-thiogalactopyranoside, the cells were further cultured for 20 h at 18 °C. The cells were harvested by centrifugation and lysed by sonication in buffer A (20 mM Tris-HCl at pH 8.5, 150 mM NaCl, 10% (*v*/*v*) glycerol and 0.1 mM tris(2-carboxyethyl) phosphine hydrochloride) containing 5 mM imidazole and 1 mM phenylmethylsulfonyl fluoride. The suspension was centrifuged for 1 h, and the supernatant was filtered using a 0.22 μm syringe filter. The filtrate was applied onto a HiTrap Chelating HP column (GE Healthcare, Chicago, IL, USA), which was pre-equilibrated with buffer A containing 5 mM imidazole. Upon applying a linear gradient of imidazole in the same buffer, PPARγ WT LBD was eluted at 50–100 mM imidazole concentration. The eluent was desalted in buffer A using a HiPrep 26/10 column (GE Healthcare, Chicago, IL, USA) before cleaving the purified protein with 2 units of thrombin (Merck Millipore, Darmstadt, Germany) per mg of PPARγ LBD. After cleavage with thrombin, both the N-terminal fusion tag and the uncleaved protein were removed on a HiTrap Chelating HP column (GE Healthcare, Chicago, IL, USA). The flow-through was applied to a HiLoad XK-16 Superdex 200 prep-grade column (GE Healthcare, Chicago, IL, USA), which was pre-equilibrated with buffer A. The purified PPARγ WT LBD was pooled and concentrated to 15.6 mg/mL using an Amicon Ultra-15 Centrifugal Filter Unit (Merck Millipore, Darmstadt, Germany). The human PPARγ R288A mutant LBD was expressed and purified in the same manner as the WT protein.

### 4.3. Crystallization

The purified PPARγ R288A mutant LBD and the coactivator SRC-1 peptide containing the LxxLL motif were mixed in a molar ratio of 1:2, in the presence or absence of a 7-fold molar excess of the PPARγ phosphorylation site inhibitor imatinib. After 24 h, the protein complexes were crystallized at 23 °C using the sitting-drop vapor diffusion method by mixing 0.2 μL of the purified protein solution and 0.2 μL of the reservoir solution. Crystals of imatinib-bound PPARγ R288A mutant LBD and ligand-free PPARγ R288A mutant LBD were obtained with a reservoir solution of 2.2 M sodium malonate at pH 7.0.

### 4.4. X-ray Data Collection

X-ray diffraction data for imatinib-bound PPARγ R288A mutant LBD were collected at 100 K using a Dectris PILATUS 2M-F pixel detector (Dectris Ltd., Baden, Switzerland) at the NE3A experimental station of Photon Factory, Japan. The X-ray data from the crystal of ligand-free PPARγ R288A mutant LBD were collected at 100 K using a Quantum Q270 CCD detector system (Area Detector Systems Corporation, Poway, CA, USA) at the BL-7A experimental station of Pohang Light Source, Korea. Raw X-ray diffraction data were processed and scaled using the program suit *HKL2000* [36]. Crystals of imatinib-bound PPARγ R288A mutant LBD belonged to the space group *P2_1_2_1_2*, with unit cell parameters of a = 130.9 Å, b = 52.8 Å, c = 53.4 Å. One monomer was present in the asymmetric unit, generating a Matthew’s parameter and solvent fraction of 2.70 Å^3^/Da and 54.5%, respectively. Crystals of ligand-free PPARγ R288A mutant LBD belonged to the space group *P2_1_2_1_2*, with unit cell parameters of a = 131.5 Å, b = 52.7 Å, c = 53.7 Å. One monomer was present in the asymmetric unit, generating a Matthew’s parameter and solvent fraction of 2.72 Å^3^/Da and 54.9%, respectively. Data collection statistics are summarized in Table 1.

### 4.5. Structure Determination and Refinement

Both structures of imatinib-bound and ligand-free PPARγ R288A mutant LBD were solved by the molecular replacement method using the program *MolRep* with the ligand-free PPARγ WT LBD structure (PDB ID: 6JQ7) as a search model [35,37]. Subsequent model building was performed manually using the program *COOT* [38]. Following this, the model was further refined using the program *REFMAC5*, including the bulk solvent correction [39]. A total of 5% of the data were randomly excluded for the calculation of *R_free_* [40]. The reliability of the refined models was assessed using *MolProbity* [41]. Refinement statistics are summarized in Table 2.

### 4.6. Surface Plasmon Resonance

The binding affinities of PPARγ WT LBD and R288A mutant LBD with imatinib were investigated by SPR kinetics experiments. All SPR measurements were performed at 25 °C using the Biacore T200 apparatus (GE Healthcare, Chicago, IL, USA). For immobilization, the amine coupling kit containing 0.1 M N-hydroxysuccinimide and 0.4 M 1-ethyl-3-(3-dimethylaminopropyl) carbodiimide hydrochloride on a CM5 sensor chip with HBS-EP buffer (10 mM HEPES at pH 7.5, 150 mM NaCl, 3 mM EDTA, and 0.005% p20) was used according to the manufacturer’s protocol. Subsequently, 30 μg/mL of PPARγ WT LBD or R288A mutant LBD dissolved in 10 mM sodium acetate at pH 5.0 was injected at regular intervals until the immobilization level reached approximately 7000 response units (RU). The remaining activated carboxyl groups on the CM5 sensor chip surface were deactivated with 1 M ethanolamine at pH 8.5 for 400 s. The control experiment was treated identically with a reference flow cell without protein, and the response by the control was subtracted from each sample dataset. Imatinib at concentrations of 0.625, 1.25, 2.50, 5.00, and 10.0 μM in PBS buffer was injected over the PPARγ WT LBD chip or R288A mutant LBD chip at a rate of 30 μL/min for 120 s, followed by dissociation for 300 s in multi-cycle reactions. For rosiglitazone, experiments were conducted at lower concentrations than imatinib. The sensor chip surface was regenerated for 5 s with 5 mM NaOH between cycles. The SPR response data were fit to the simple bimolecular 1:1 Langmuir isotherm binding model to determine the equilibrium dissociation constant (*Kd*) using Biacore T200 evaluation software 3.0 (GE Healthcare, Chicago, IL, USA).

### 4.7. Accession Codes

Atomic coordinates and structure factors for ligand-free and imatinib-bound PPARγ R288A mutant LBD structures have been deposited in Protein Data Bank under the accession codes 6KTM and 6KTN, respectively.

## Figures and Tables

**Table 1:** Statistics for the data collection.

Model Name	Imatinib-Bound (PDB ID: 6KTN)	Ligand-Free (PDB ID: 6KTM)
X-ray source	PF-NE3A	PLS-7A
X-ray wavelength (Å)	1.00000	0.97933
Space group	*P2_1_2_1_2*	*P2_1_2_1_2*
Unit cell parameters		
a, b, c (Å)	130.93, 52.78, 53.44	131.47, 52.70, 53.67
α = β = γ (°)	90	90
Resolution range (Å)	50.0–2.75 (2.80–2.75) ^a^	50.0–2.70 (2.75–2.70) ^a^
Total/unique reflections	69,349/10,176	101,468/10,775
Completeness (%)	99.9 (99.6) ^a^	99.6 (100.0) ^a^
*<I/σI>*	30.1 (2.7) ^a^	41.1 (4.1) ^a^
*R_merge_*^b^ (%)	5.9 (67.9) ^a^	7.9 (70.4) ^a^
CC_1/2_	0.971 (0.860) ^a^	0.975 (0.875) ^a^

^a^ Values in parentheses refer to the highest resolution shell. ^b^ *R_merge_* = ΣhΣi|I(h)i–<I(h)>|/ΣhΣi I(h)i, where I(h) is the intensity of reflection h, Σh is the sum over all reflections, and Σi is the sum over i measurements of reflection h.

**Table 2:** Statistics for the model refinement.

Model Name	Imatinib-Bound	Ligand-Free
Resolution range (Å)	30.0–2.75	30.0–2.70
*R*_work_/*R*_free_^a^ (%)	22.1/25.6	21.7/25.8
No. of nonhydrogen atoms		
Protein	2216	2174
Ligand	37	-
Water oxygen	30	20
Average *B* factor (Å^2^)		
Protein	48.3	77.9
Ligand	56.1	-
Water oxygen	38.3	60.4
R.m.s. deviations from ideal geometry		
Bond lengths (Å)	0.007	0.005
Bond angles (°)	1.39	1.31
Ramachandran plot ^b^		
Favored/Outliers (%)	93.3/0.0	98.5/0.0
Poor rotamers ^b^ (%)	0.00	0.00

^a^*R*_work_ = Σ||*F*_obs_| – |*F*_calc_||/Σ |*F*_obs_|, where *R*_free_ is calculated for a randomly chosen 5% of reflections, which were not used for structure refinement and *R*_work_ is calculated for the remaining reflections. ^b^ Values obtained using *MolProbity*.

## Supplementary Materials

The following are available online. Figure S1: Superposition of imatinib-bound ABL kinase domain (PDB ID: 1IEP) and imatinib-bound Syk kinase domain (PDB ID: 1XBB), Figure S2: Ribbon diagram of the H9–H10 loop region in the ligand-free PPARγ WT LBD structure (PDB ID: 6JQ7), Figure S3: The hydrophobic interaction network in Arm3 region of PPARγ WT LBD, Figure S4: SPR analysis of the binding affinity for rosiglitazone of PPARγ WT LBD, Figure S5: Modeling of the ligand-free PPARγ WT LBD structure into the imatinib-bound PPARγ R288A mutant LBD structure.
