# Supplementary material for: Structural Basis for the Regulation of PPARγ Activity by Imatinib
Source: Molecules. 2019 Oct 1;24(19):3562. doi: 10.3390/molecules24193562 (PMC6803859; doi:10.3390/molecules24193562)
Supplement: Supplementary file 1 [file molecules-24-03562-s001.pdf]

molecules

MDPI

Supplementary materials for

Structural basis for the regulation of PPARγ activity by imatinib

Jun Young Jang[1], Hyun-Jung Kim[2], and Byung Woo Han[1*]

[1] Research Institute of Pharmaceutical Sciences, College of Pharmacy, Seoul National University, Seoul, Korea

[2] College of Pharmacy, Chung-Ang University, Seoul, Korea

* Correspondence: bwhan@snu.ac.kr; Tel.: +82-02-880-7898

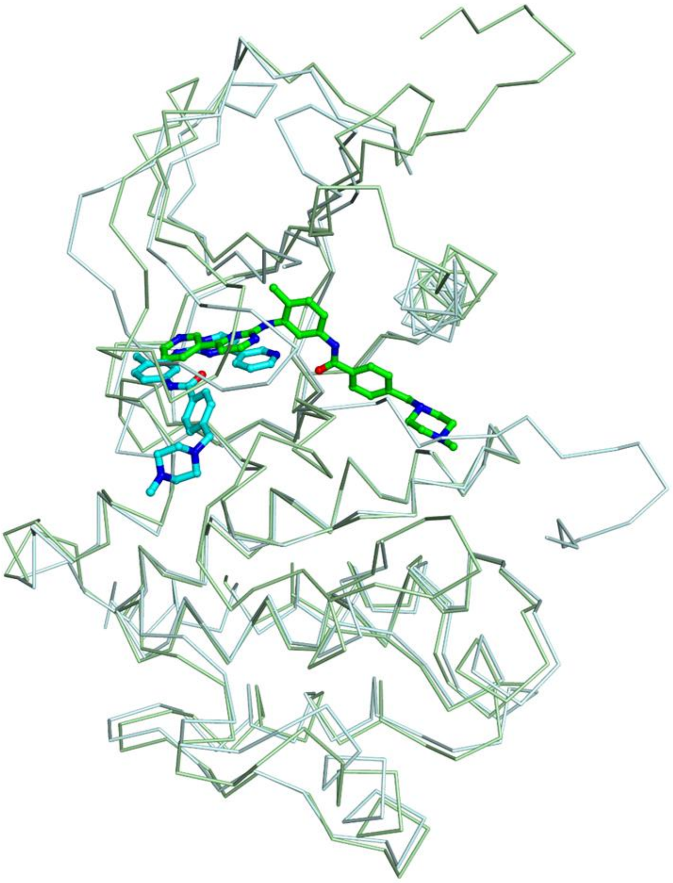

Figure S1. Superposition of imatinib-bound ABL kinase domain (PDB ID: 1IEP) and imatinib-bound Syk kinase domain (PDB ID: 1XBB). ABL kinase domain (pale green) and Syk kinase domain (pale cyan) are shown in Cα trace ribbon models. Imatinib molecules in the ABL and Syk complex structures are shown as green and cyan stick models, respectively.

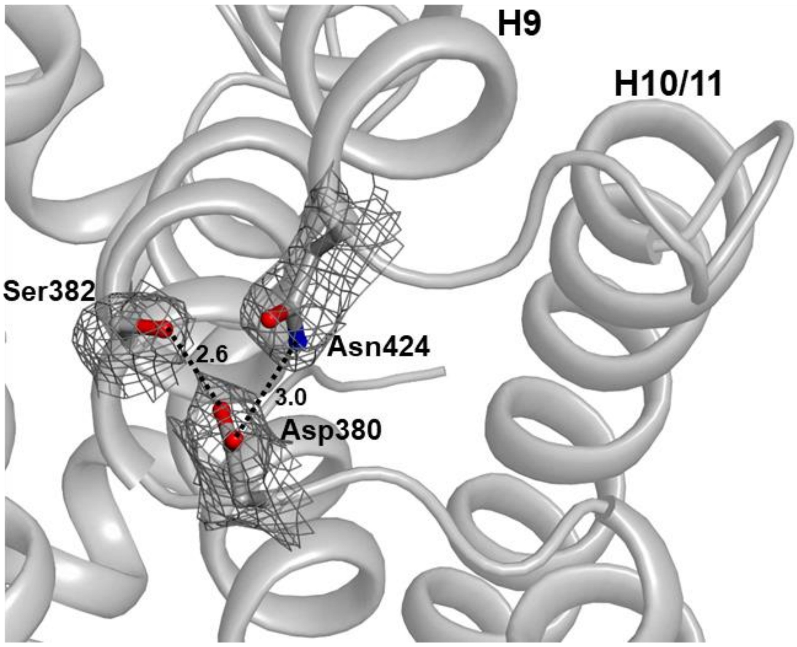

Figure S2. Ribbon diagram of the H9-H10 loop region in the ligand-free PPARγ WT LBD structure (PDB ID: 6JQ7). The residues Asp380, Ser382, and Asn424 are shown in stick models with *2mFo-DFc* electron density maps (in grey; contoured at 1.0σ). Dashed lines represent the distance between residues and the corresponding distances (Å) are labeled.

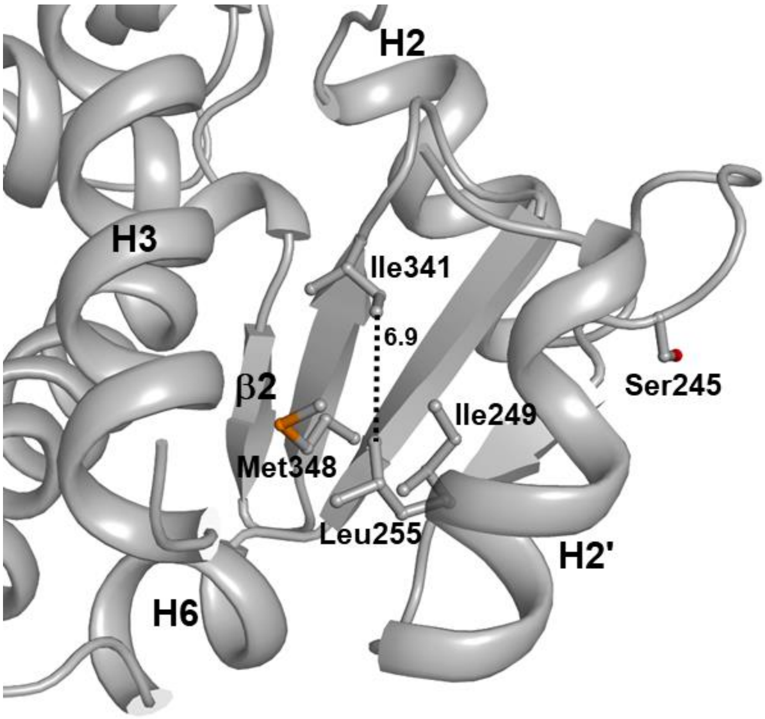

Figure S3. The hydrophobic interaction network in Arm3 region of PPARγ WT LBD. The ligand-free PPARγ WT LBD structure is shown in a ribbon diagram (grey), and the residues forming the hydrophobic interaction network are represented by stick models. Dashed line represents the distance between Leu255 and Ile341, and the corresponding distances (Å) are labeled.

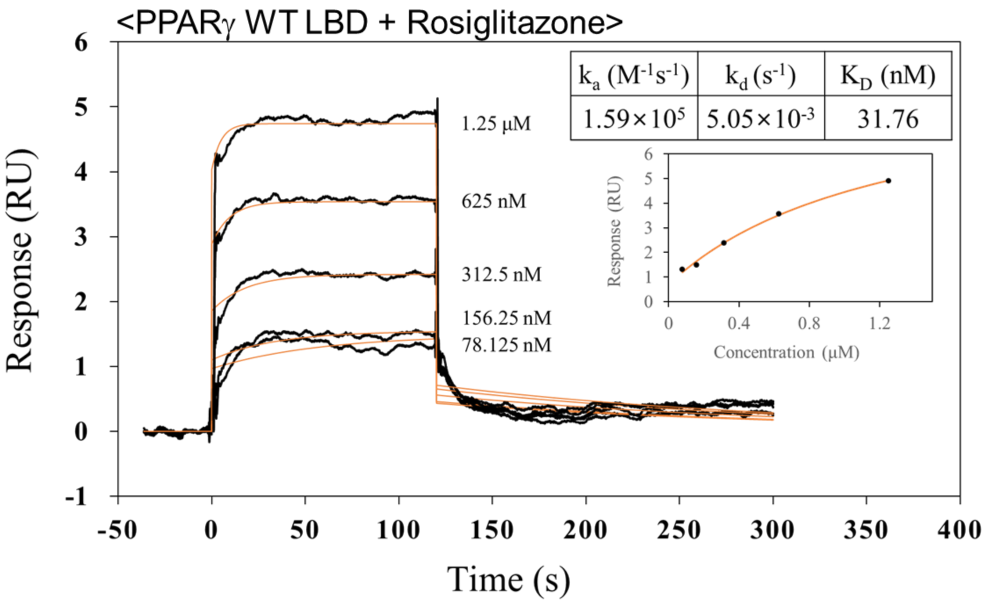

Figure S4. SPR analysis of the binding affinity for rosiglitazone of PPARγ WT LBD. SPR sensorgrams for rosiglitazone binding of PPARγ WT LBD are shown. The calculated Kd value is also shown.

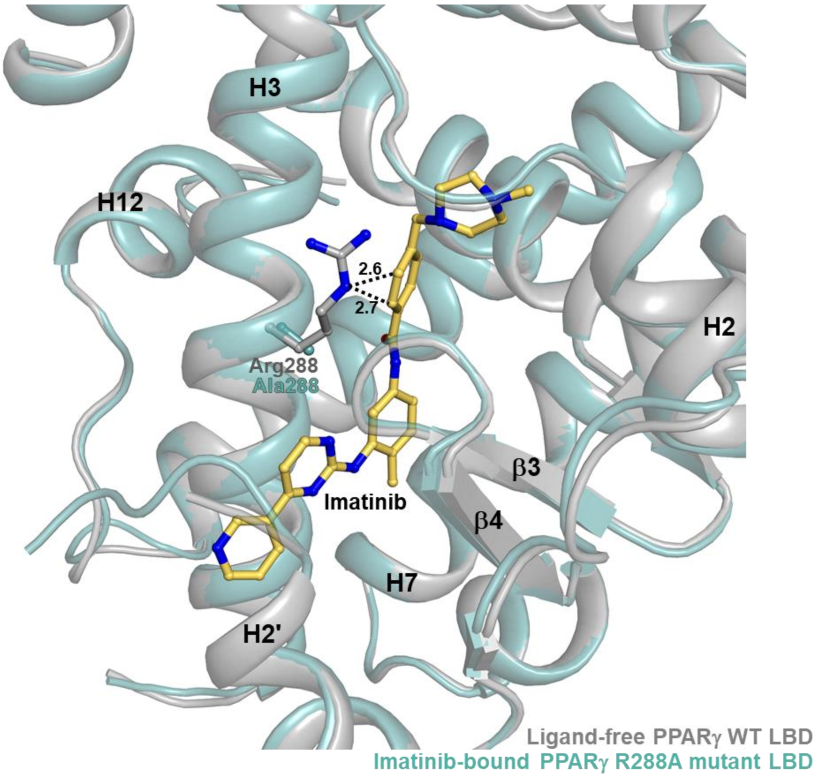

Figure S5. Modeling of the ligand-free PPARγ WT LBD structure into the imatinib-bound PPARγ R288A mutant LBD structure. The ligand-free PPARγ WT LBD structure (PDB ID: 6JQ7) was superimposed onto the imatinib-bound PPARγ R288A mutant LBD structure. The ligand-free PPARγ WT and the imatinib-bound PPARγ R288A mutant LBD structures are shown in grey and light teal colored ribbon diagrams, respectively. Side chains and imatinib are shown as stick models. Dashed line represents the distance between Arg288 and imatinib, and the corresponding distances (Å) are labeled.

© 2019 by the authors. Submitted for possible open access publication under the terms and conditions of the Creative Commons Attribution (CC BY) license (http://creativecommons.org/licenses/by/4.0/).
